# The good, the bad, and the timely: how temporal order and moral judgment influence causal selection

**DOI:** 10.3389/fpsyg.2014.01336

**Published:** 2014-11-18

**Authors:** Kevin Reuter, Lara Kirfel, Raphael van Riel, Luca Barlassina

**Affiliations:** ^1^Department of Philosophy, Institute of Philosophy II, Ruhr University BochumBochum, Germany; ^2^Department of Philosophy, King’s College LondonLondon, UK; ^3^Faculty of Humanities, Institute for Philosophy, University of Duisburg-EssenEssen, Germany; ^4^Department of Philosophy, University of SheffieldSheffield, UK

**Keywords:** causation, causal selection, temporal location, moral judgment, norm violation

## Abstract

Causal selection is the cognitive process through which one or more elements in a complex causal structure are singled out as actual causes of a certain effect. In this paper, we report on an experiment in which we investigated the role of moral and temporal factors in causal selection. Our results are as follows. First, when presented with a temporal chain in which two human agents perform the same action one after the other, subjects tend to judge the later agent to be the actual cause. Second, the impact of temporal location on causal selection is almost canceled out if the later agent did not violate a norm while the former did. We argue that this is due to the impact that judgments of norm violation have on causal selection—even if the violated norm has nothing to do with the obtaining effect. Third, moral judgments about the effect influence causal selection even in the case in which agents could not have foreseen the effect and did not intend to bring it about. We discuss our findings in connection to recent theories of the role of moral judgment in causal reasoning, on the one hand, and to probabilistic models of temporal location, on the other.

## INTRODUCTION

Mary is filling up her car at a gas station. It was a tough week: she is extremely tired. Inadvertently, she spills a good amount of gasoline on the ground, then she walks to the counter. In that moment, John arrives. He knows that it is prohibited to smoke at a gas station, but he does not care. As soon as he throws the cigarette on the ground, the gasoline spilled by Mary catches fire, and Mary’s car explodes. Who caused the explosion? When you are asked this question, you are faced with a problem of *causal selection*: you have to single out which element(s) in a complex *causal structure* is(are) the *actual cause*(s) of a certain effect. Who are you going to choose: Mary, John, both, or neither? You should be careful here. Something bad happened: a car exploded. Someone could have been seriously injured, even killed. Thus, the person that you pick out to be the actual cause is going to face serious trouble.

So, what’s your pick? There are several elements that can guide your choice. To begin with, there is a *temporal difference* between Mary’s and John’s actions: the former took place before the latter. Moreover, John *intentionally* dropped the cigarette, while Mary spilled the gasoline accidentally. Finally, John *violated a rule*. Which of these factors, if any, will guide your judgment of actual causation? And how will these factors interact? If you want to know about this (and you *ought* to, given the tremendous consequences that a judgment of actual causation might have), you might want to read this paper. In it, we report on an experiment in which we investigated how moral and temporal factors influence causal selection.

*Temporal location* is the temporal position that an element occupies in a certain causal structure. In the previous story, for example, Mary’s spilling gasoline and John’s throwing the cigarette had different temporal locations, since the former occupied an earlier temporal position than the latter. Many studies have shown that temporal location is of crucial importance in causal selection. However, while some scholars have argued that people tend to single out the *initiating event* in a causal structure to be the actual cause (Vinokur and Ajzen, 1982; Johnson et al., 1989), other researchers have proposed that causal selection processes favor the *last event* just before the outcome (Einhorn and Hogarth, 1986; N’gbala and Branscombe, 1995).

Spellman (1997) has tried to reconcile these opposite perspectives by suggesting that the effect of temporal location on causal selection can be traced back to probability raising: people select as actual cause the element in a causal structure that raises the probability of the effect the most. This explains why people identify the last event to be the actual cause in *temporal chains*, i.e., in causal structures in which successive events are causally independent of each other^1^, but identify the initial event to be the actual cause in *unfolding chains*, i.e., in causal structures in which later events causally depend on earlier events (Miller and Gunasegaram, 1990)^2^. Here is an intuitive example of a temporal chain: the fish owned by a family needs to be fed once a day. If it gets overfed, it dies. In the morning, the mother feeds it. In the afternoon, the kids feed it. In the evening, the father feeds it. The fish dies. In contrast, the domino effect would be an instance of an unfolding chain: the first toppling tile causes the next to fall, which, in turn, causes the next to fall, etc.

It has been recently argued, however, that probabilistic models of the role of temporal location in causal selection fall short of accounting for causal reasoning about human actions. For example, Hilton et al. (2005, 2009) have shown that people tend to favor human actions over physical events as actual causes, irrespective of temporal location and change in probability, while McClure et al. (2007) have established that, when presented with an *opportunity chain*, i.e., a causal structure in which an earlier event creates the opportunity for a second event to occur^3^, people judge the latter event to be the actual cause if both events are physical, but judge both events to be actual causes if both events are human actions. The following case serves as a paradigmatic example of an opportunity chain: a man lighting a fire in the forest makes it possible for a gust of wind to spread smoke across the whole forest.

These findings suggest that there might be a difference between the principles guiding causal selection in the case of purely physical events and those guiding causal selection in the case of human actions. In particular, while causal reasoning about physical events could be entirely guided by statistical information, causal reasoning about human actions would appear to be sensitive to other elements as well. Which other elements? Recently, Knobe (2010) has put forward the intriguing hypothesis that, in the case of human actions, causal selection is influenced by moral considerations. This brings us to the second main component of our study.

Consider the so-called *Pen Case* (Knobe and Fraser, 2008):

The receptionist in the philosophy department keeps her desk stocked with pens. The administrative assistants are allowed to take the pens, but faculty members are supposed to buy their own.

The administrative assistants typically do take the pens. Unfortunately, so do the faculty members. The receptionist has repeatedly emailed them reminders that only administrative assistants are allowed to take the pens.

On Monday morning, one of the administrative assistants encounters Professor Smith walking past the receptionist’s desk. Both take pens. Later that day, the receptionist needs to take an important message…but she has a problem. There are no pens left on her desk.

When asked who caused the problem that there were no pens left, participants answered that Professor Smith caused it to a significantly higher degree than they answered that the administrative assistant caused it. Apparently, this pattern of answers cannot be accounted for in terms of statistical information, given that Professor Smith’s behavior and the assistant’s have the same typicality degree and raise the probability of the effect to the same extent. On the other hand, since there is an important moral difference between what Professor Smith and the administrative assistant did—the former, but not the latter, did something *wrong*—, it is plausible to hypothesize that subjects’ causal selection processes were influenced by moral judgments. But what exactly was wrong with Professor Smith’s behavior? On the one hand, it was wrong because it was a *norm violation*; on the other hand, it was wrong because it resulted in a *bad effect*. Thus, the following question arises: what is the respective contribution of *moral judgments of norm violation* and *moral judgments about the goodness/badness of the effect* in causal selection? In recent years, this question has received two main, competing answers.

The Norm Violation Account (NVA; Hitchcock and Knobe, 2009) maintains that the only moral judgments that impact on causal selection are moral judgments of norm violation. NVA has it that causal selection is sensitive to normality: if a subject judges an element in a causal structure to be abnormal, she will tend to select it as the actual cause. Violations of moral norms are abnormal events^4^. Thus, NVA predicts that if a subject judges that a certain element in a causal structure constitutes a violation of a moral norm, then she will tend to select it as the actual cause. Accordingly, NVA explains the Pen Case as follows: subjects singled out Professor Smith as the actual cause because Professor Smith’s behavior, but not the administrative assistant’s behavior, was counternormative.

The culpable control model (CCM; Alicke, 2000) instead proposes that both moral judgments of norm violation and moral judgments about the effect impact on causal selection, and that they both do so through a process of *blame validation*. Suppose that S performed action A and effect E followed. According to CCM, two processes are involved in evaluating what S did. On the one hand, there is a deliberative, rational process that evaluates whether S intentionally performed A, whether A caused E, and whether S foresaw E. On the other hand, there is a spontaneous, affective process that evaluates the moral status of S, A, and E (for example, whether S had malicious intentions, whether A was a violation of a norm, and whether E was a good or bad outcome). If these spontaneous moral evaluations are sufficiently negative to trigger a blame attribution to S, then the elements evaluated by the deliberative process (i.e., intentionality, causality, and foreseeability) get processed in a “blame validation mode”: in order to validate their desire to blame S, people exaggerate the extent to which S intentionally did A, or the extent to which A caused E, or the extent to which S foresaw E. Hence, CCM explains the Pen Case as follows: the fact that Professor Smith violated a norm and that a bad outcome occurred generated in the subjects a desire to blame Professor Smith; thus, to validate their desire to blame Professor Smith, subjects heightened Professor Smith’s causal role.

Both NVA and CCM are interesting accounts of the role of moral judgment in causal selection. However, given that only a few experiments on the relation between morality and causation have been so far conducted, it is not possible yet to adjudicate which of these accounts, if any, is the right one. In particular, further experimental investigation is required to disentangle the *distinctive contributions* to causal selection of moral judgments of norm violation and moral judgments about the effect. First, extant experimental scenarios are such that the violated norm is always intimately connected with the effect that obtains (Knobe and Fraser, 2008; Hitchcock and Knobe, 2009; Alicke et al., 2011). Accordingly, we currently lack data concerning how moral judgments about the effect and moral judgments of norm violation contribute to causal selection when norms and effects are independent of each other. Second, in the great majority of the experiments so far conducted on the role of moral judgments about the effect in causal selection, the agent intended to bring about the effect or was in a position to foresee that it would occur (a notable exception is Alicke, 1992). Hence, the currently available evidence does not establish how strongly causal selection is shaped by moral judgments about the goodness or badness of the effect *per se* and how strongly it is affected by folk psychological judgments about the foreseeability of the effect.

The experiment we report in this paper had four main aims. The *first* one was to expand on the empirical research on the role of temporal location in causal selection. In particular, we were interested in whether subjects, if presented with a *temporal chain* in which two human actions *differ only in their temporal location*, would judge the later action to be the actual cause, as it is the case for temporal chains involving purely physical events (Einhorn and Hogarth, 1986; Miller and Gunasegaram, 1990; N’gbala and Branscombe, 1995), or would consider both actions to be actual causes, as is the case for opportunity chains involving human actions (McClure et al., 2007). The *second* and *third* aims of our study concerned the role of morality in causal selection. On the one hand, we were interested in how moral judgments about the effect impact on causal selection, independently of folk psychological attributions of intentionality and foreseeability (see Shultz and Wright, 1985 for a related study on the concept of neglect); on the other hand, we wanted to assess the roles of moral judgments of norm violation and moral judgments about the effect in causal selection when norms and effects are entirely independent of each other. Finally, the *fourth* aim was to explore an important but entirely overlooked issue, i.e., the interplay between morality and temporality in causal selection.

## MATERIALS AND METHODS

### PARTICIPANTS

Two thousand twenty-two participants registered with Amazon’s Mechanical Turk (MTURK) website were recruited. The sample consisted of 846 females, 1165 males, and 11 people who did not identify or did not want to be identified with either gender. The mean age of our sample was 30.51 (SD = 9.89, age_min = 18). Individuals who indicated that their mother tongue was not English or did not fill out the survey completely were excluded. Participants were also asked about the amount of philosophical training they had received in the past: None = 396, Some informal reading = 376, Some undergraduate classes = 732, Completed BA = 398, Current MA student = 28, Completed MA = 72, Current PhD student = 10, Completed PhD = 10. A statistical analysis revealed neither significant differences between the responses of females and males, nor significant differences between the responses of participants who had some philosophical training and those who did not.

### DESIGN AND PROCEDURES

We started from the following *synchronous, neutral effect, no-norm* scenario involving a *temporal chain* (Scenario 1)^5^:

Alice and Zoe work for the same company. They work in different rooms and both of them sometimes need to access the central computer of the company. Unbeknownst to everybody, if two people are logged in to the central computer at the same time, an empty email is immediately sent from the central computer to a non-existent email address.

One day, Alice logs in to the central computer at 9 am. The same day, Zoe also logs in at 9 am. Immediately, an empty email is sent from the central computer to a non-existent email address.

We then manipulated this scenario along three dimensions: (A) temporal location (synchronous, Alice first, Zoe first); (B) moral status of the effect (neutral, good, bad)^6^; (C) norm violation (no norm, Zoe violates a norm), thus obtaining the following 3 × 3 × 2 experimental design (**Table 1**):

**Table 1:** Labeling structure of the scenarios that were presented to subjects given the 3 × 3 × 2 experimental design.

	No norm			Norm violation		
	Synchronous	Alice first	Zoe first	Synchronous	Alice first	Alice first
Neutral effect	1	2	3	10	11	12
Good effect	4	5	6	13	14	15
Bad effect	7	8	9	16	17	18

To illustrate, here are a few scenarios:

*Alice first, good effect, no norm* (Scenario 5)

Alice and Zoe work for the same company. They work in different rooms and both of them sometimes need to access the central computer of the company. Unbeknownst to everybody, if two people are logged in to the central computer at the same time, some spam e-mails containing dangerous viruses are immediately deleted from the central computer.

One day, Alice logs in to the central computer at 9 am. The same day, Zoe logs in at 9:30 am, when Alice is already logged in. Immediately, some spam e-mails containing dangerous viruses are deleted from the central computer.

*Zoe first, bad effect, Zoe violates a norm* (Scenario 18)

Alice and Zoe work for the same company. They work in different rooms and both of them sometimes need to access the central computer of the company. Unbeknownst to everybody, if two people are logged in to the central computer at the same time, some work e-mails containing important customer information are immediately deleted from the central computer.

In order to make sure that one person is always available to answer incoming phone calls, the company issued the following official policy: Alice is the only one permitted to log in to the central computer in the mornings, whereas Zoe is the only one permitted to log in to the central computer in the afternoons.

One day, violating the official policy, Zoe logs in to the central computer at 9 am. The same day, following the official policy, Alice logs in at 9.30 am, when Zoe is already logged in. Immediately, some work e-mails containing important customer information are deleted from the central computer.

Each participant received only one of these 18 scenarios and was either asked a single forced-choice question about causal attribution (Q1: 1283 individuals) or two questions in randomized order regarding the blameworthiness/praiseworthiness of certain acts described by the scenario (Q2 and Q3: 739 individuals). In order to make sure that no person had already been acquainted with one of the scenarios before, participants who answered more than one vignette were excluded.

Q1. Depending on the scenario the subject was presented with, s/he answered one of the following questions:

*Neutral:* Who caused an empty email to be sent from the central computer to a non-existent email address?
*Bad*: Who caused some work e-mails containing important customer information to be deleted from the central computer?
*Good*: Who caused some spam e-mails containing dangerous viruses to be removed from the central computer?

(Subjects had to choose one among the following five answers: (i) Alice, (ii) Zoe, (iii) Both, (iv) None of the two, (v) Not sure).

Q2: How would you evaluate Alice’s logging in to the computer, on a scale from ‘-3’ to ‘3’ where ‘-3’ means ‘Very blameworthy,’ ‘0’ means ‘Neither blameworthy nor praiseworthy’ and ‘3’ means ‘Very praiseworthy’?

Q3: How would you evaluate Zoe’s logging in to the computer, on a scale from ‘-3’ to ‘3’ where ‘-3’ means ‘Very blameworthy,’ ‘0’ means ‘Neither blameworthy nor praiseworthy’ and ‘3’ means ‘Very praiseworthy’?

Our methodology departs from most other studies in that subjects are normally requested to say *how much* a certain element in a causal chain contributed to a certain effect (e.g., Lagnado and Channon, 2008; Hilton et al., 2009). We chose not to use *graded questions* (e.g., ‘How much did X contribute to E?’), but rather ungraded *who-questions* because while the latter are normally read as asking for a *causal report* about what happened in the world, the former are systematically ambiguous, since they can be interpreted either as asking for a causal report or as asking for a *causal explanation* (the distinction between causal reports and causal explanations is inspired by Davidson (1967), Beebee (2004), and Varzi (2007). Since our goal was to investigate the impact of temporal and moral considerations on causal judgments rather than on causal explanations, we decided to use ungraded who-questions rather than graded questions^7^. A second reason to favor who-questions to questions like ‘How much did X contribute to E?’ is that we were interested in judgments of actual causation, but the notion of contribution is not sensitive to the distinction between actual causes and enabling conditions. In other words, a sentence like ‘X contributed to E’ can be true even if X is merely an enabling condition, and not an actual cause, of E. For example, it is true that gravity (an enabling condition, but not an actual cause) always contributes to a plane crash (see, e.g., Cheng and Novick, 1991).

## RESULTS

### MULTINOMIAL LOGISTIC REGRESSION

Multinomial logistic regression was applied to integrate the three independent categorical variables *temporal location*, moral status of the *effect*, and *norm violation* in a statistical model designed to identify predictors of causal attribution. All analyses were performed with SPSS Statistics 22. Two models showed a high fit to the data according to fit indices Pearson χ^2^, Pseudo R^2^ (Cox & Snell) and Likelihood Ratio Test. First, all independent factors plus an interaction between *norm violation* and *temporal order* yield Pearson (χ^2^ = 46.441; *p* = 0.224) and Cox & Snell = 0.490 with all factors highly significant, *p* < 0.0005. Second, an additional interaction between *norm violation* and *effect* turned out to be marginally significant *p* = 0.055, with Pearson (χ^2^ = 29.978; *p* = 0.569) and Cox & Snell = 0.496. No significant interaction between *effect* and *temporal order* was found.

Parameter estimates indicated that subjects were significantly less likely to select ‘Alice’ rather than ‘Both’ as the actual cause when Alice and Zoe logged in together (*B* = -1.364; *p* = 0.013). When Zoe logged in later, subjects were significantly more likely to select ‘Zoe’ as the actual cause rather than ‘Both’ (*B* = 0.579; *p* = 0.034); on the other hand, subjects were significantly less likely to select ‘Zoe’ rather than ‘Both’ as the actual cause when Zoe did not violate any norm (*B* = -5.173; *p* < 0.001). Finally, subjects were less likely to select ‘None of the two’ rather than ‘Both’ as the actual cause when the effect was neutral (*B* = -2.197, *p* = 0.003) or good (*B* = -1.400; *p* = 0.025).

### CAUSAL SELECTION

Fisher’s Exact Test was used to determine possible significant relationships between two scenarios in the 3 × 3 × 2 experimental design, operating with a 3 × 3 × 2 × 5 contingency table. Fisher’s Exact Test was necessary because some frequencies inside the contingency table were lower than 5. Repeating Fisher’s Exact Test for various pairwise comparisons made it necessary to adjust the level of significance. Having limited our analysis to 14 pairwise comparisons, the level of significance reduced to 0.0036 (using conservative Bonferroni correction).

#### Temporal location

**FIGURE 1:**
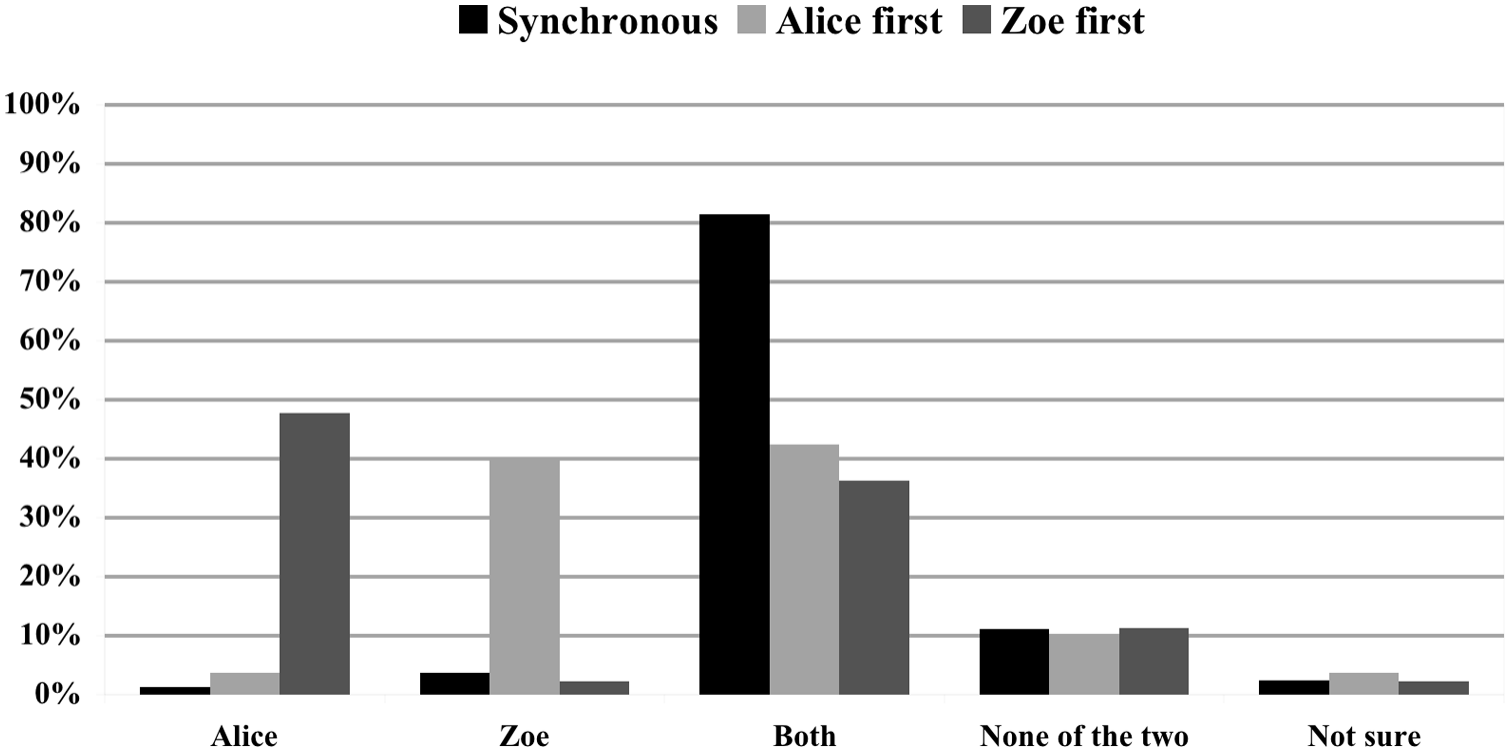
**Impact of temporal order on causal judgments.** Causal ratings in % for Scenario 1 (Alice and Zoe log in synchronously), Scenario 2 (Alice logs in first), and Scenario 3 (Zoe logs in first). The effect is held neutral and no rule violation occurs.

The first analysis provided results on how temporal location impacted on causal selection in a temporal chain when the effect was held neutral and no norm violation occurred. Three conditions were compared: Scenario 1: both Alice and Zoe log in at the same time (*N* = 81); Scenario 2: Alice logs in first (*N* = 80); Scenario 3: Zoe logs in first (*N* = 40). As can be seen in **Figure 1**, in Scenario 1 81.5% of subjects selected ‘Both’ and 11.1% of subjects opted for ‘None of the two.’ On the other hand, when either Alice or Zoe logged in first, the percentage of people who chose ‘Both’ reduced to 42.5% and 36.4% respectively, with 40.0% selecting ‘Zoe’ in Scenario 2 and 47.7% selecting ‘Alice’ in Scenario 3. The difference between Scenario 3 and Scenario 1 was significant (χ^2^ = 44.847; *p* < 0.001); however, as expected, there was no significant difference between Scenario 2 and Scenario 3 when the names Alice and Zoe are interchanged (χ^2^ = 1.153; *p* = 0.920).

#### Effect

**FIGURE 2:**
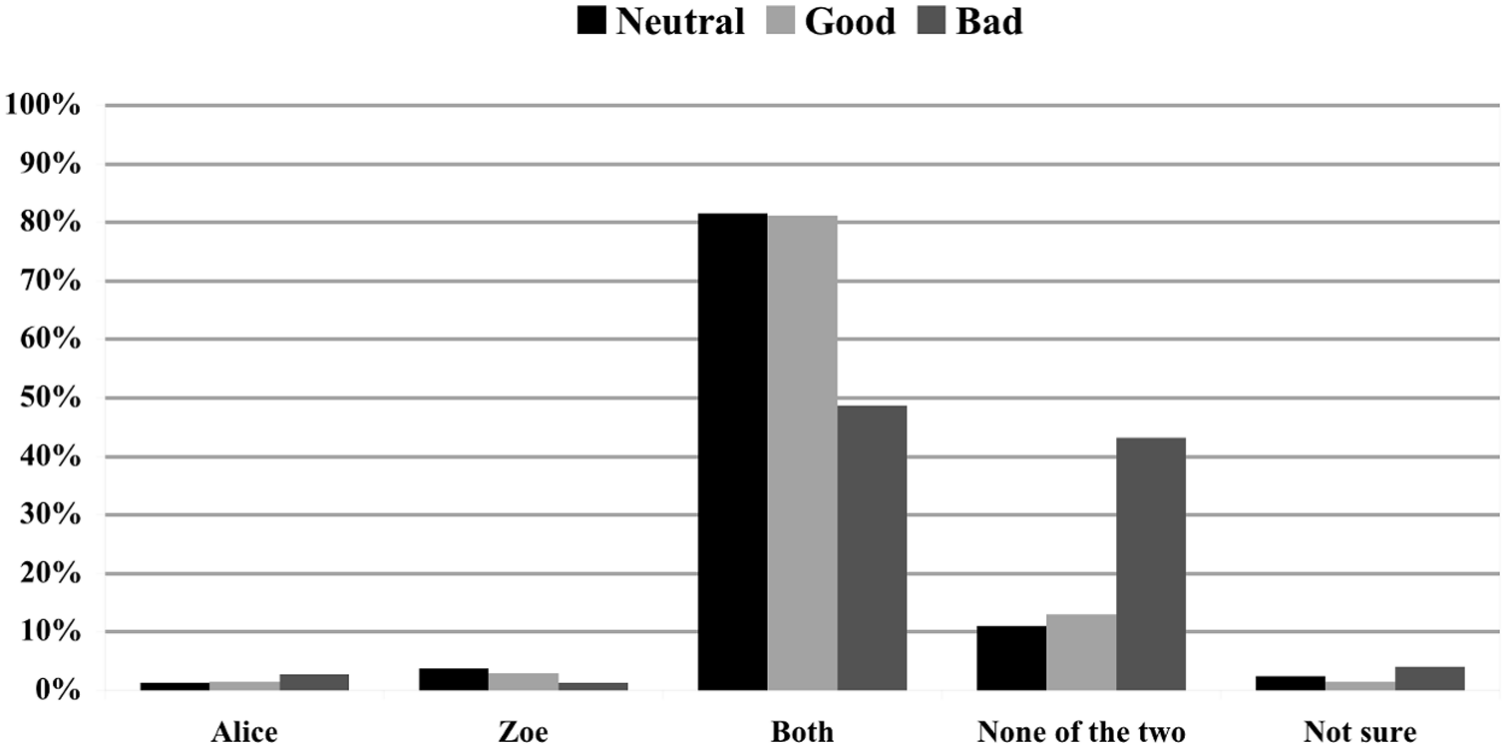
**Impact of the moral status of the effect on causal judgments.** Causal ratings in % for Scenario 1 (neutral effect), Scenario 4 (good effect), and Scenario 7 (bad effect). Alice and Zoe both log in synchronously and do not violate a rule.

The next analysis assessed how modifying the moral status of the effect impacted on causal selection when no norm was violated and the two agents acted simultaneously. As can been seen in **Figure 2**, changing the effect from neutral to good (Scenario 1 vs. Scenario 4, *N* = 69) did not have any impact on people’s causal selection process: 81.5% vs. 81.2% choosing ‘Both’ and 11.1% vs. 13% choosing ‘None of the two’—no significant difference between both conditions: (χ^2^ = 0.832; *p* = 0.987). In contrast, changing the outcome from neutral to bad (Scenario 1 vs. Scenario 7, *N* = 74) had a significant effect on people’s responses: the amount of ‘Both’ responses dropped from 81.5 to 48.6%, whereas the percentage of ‘None of the two’ responses rose from 11.1 to 43.2%, (χ^2^ = 22.990; *p* < 0.001).

#### Norm violation

**FIGURE 3:**
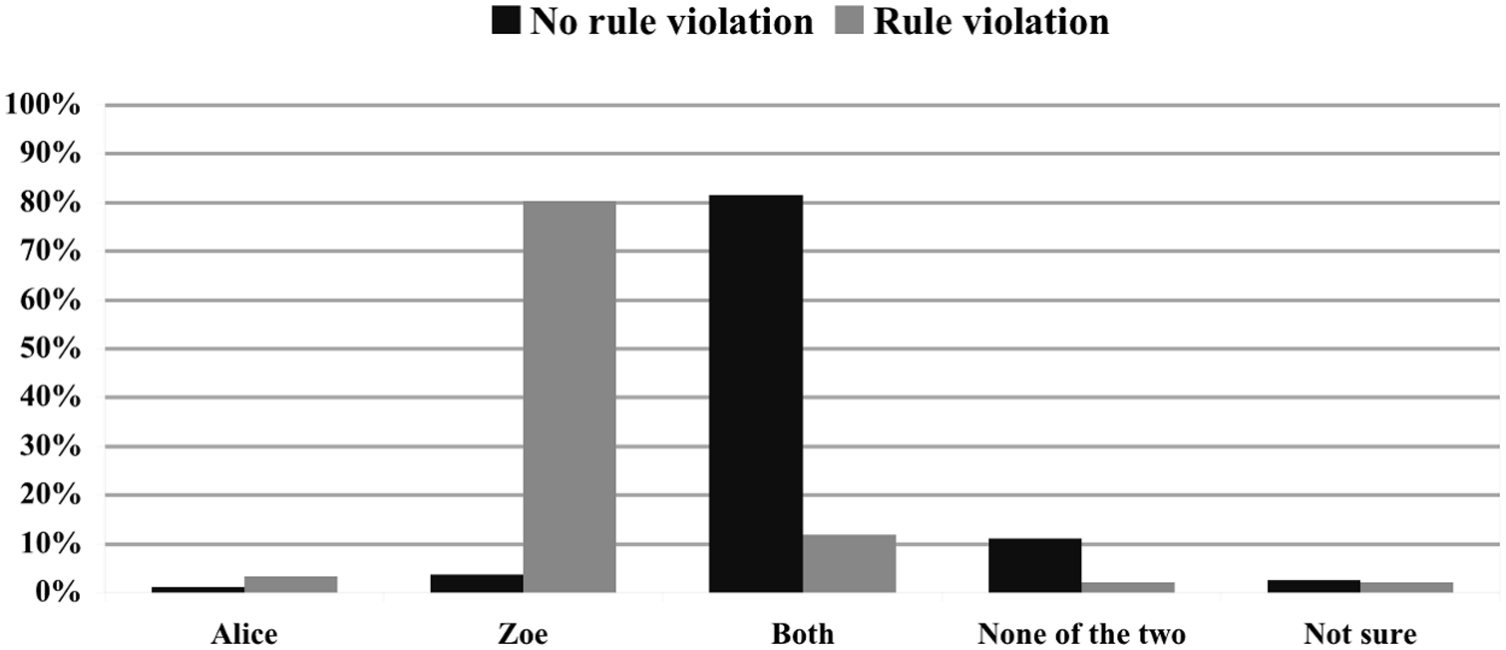
**Impact of norm violation on causal judgments.** Comparison of the causal ratings in % for Scenario 1 (no rule violation) and Scenario 10 (Zoe violates a rule). The effect is held neutral and Alice and Zoe log in synchronously.

To assess the impact of norm violation on causal selection, Scenario 1 (Alice and Zoe log in at the same time; neutral effect; no norm violation) and Scenario 10 (Alice and Zoe log in at the same time; neutral effect; Zoe violates a norm) were compared. As shown by **Figure 3**, whereas 81.5% selected ‘Both’ to be the cause in Scenario 1, 74 of 92 participants consider Zoe to be the sole cause when she violated the company policy (χ^2^ = 127.198; *p* < 0.001).

#### Effect × temporal location

**FIGURE 4:**
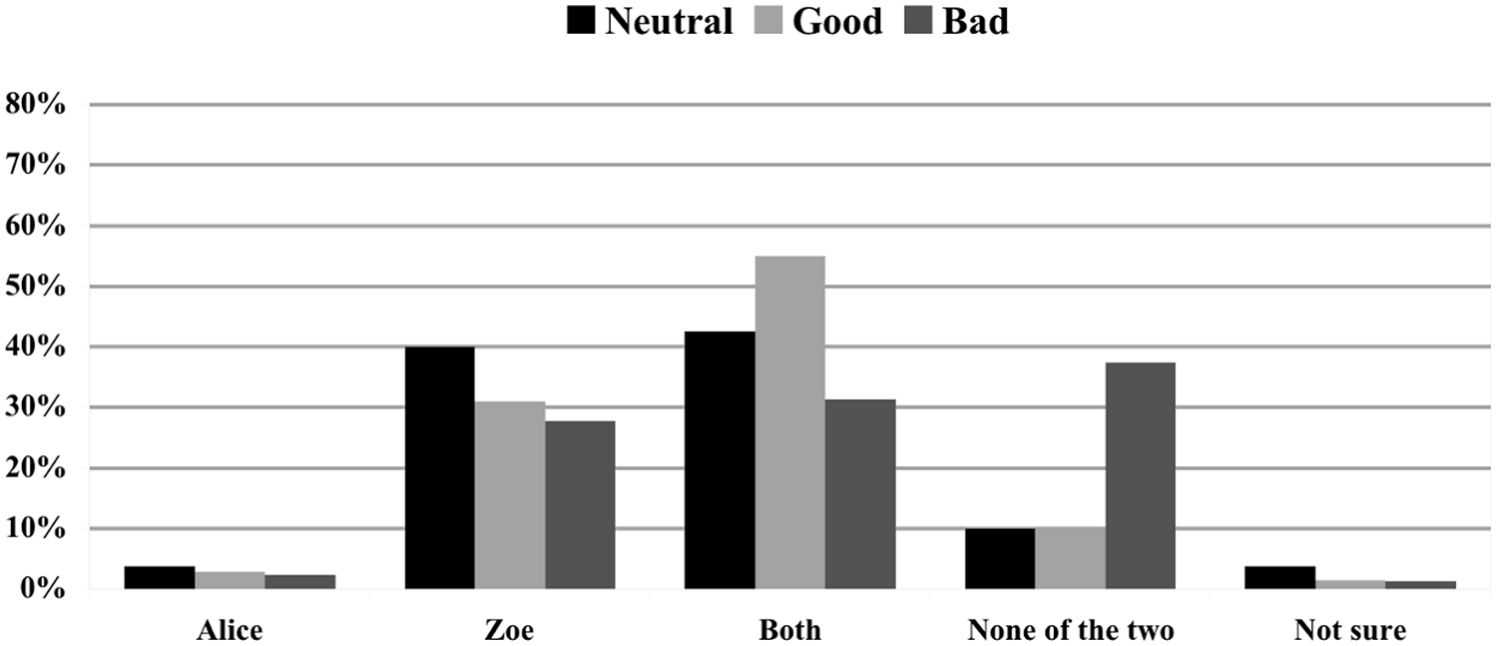
**Impact of the moral status of the effect on causal judgments in the asynchronous condition.** Causal ratings in % for Scenario 2 (neutral effect), Scenario 5 (good effect) and Scenario 8 (bad effect). No rule violation occurs.

Scenarios 2, 5, and 8 were compared to investigate how the moral status of the effect impacted on causal selection when no norm was violated and the two agents acted one after the other (**Figure 4**). Again, no significant difference between the neutral scenario (Scenario 2, *N* = 80) and the good scenario (Scenario 5, *N* = 70) was found, while a negative outcome (Scenario 8, *N* = 83) increased the percentage of the ‘None of the two’ answer from 9.9 to 37.3% (χ^2^ = 17.746; *p* = 0.001).

#### Norm violation × temporal location

**FIGURE 5:**
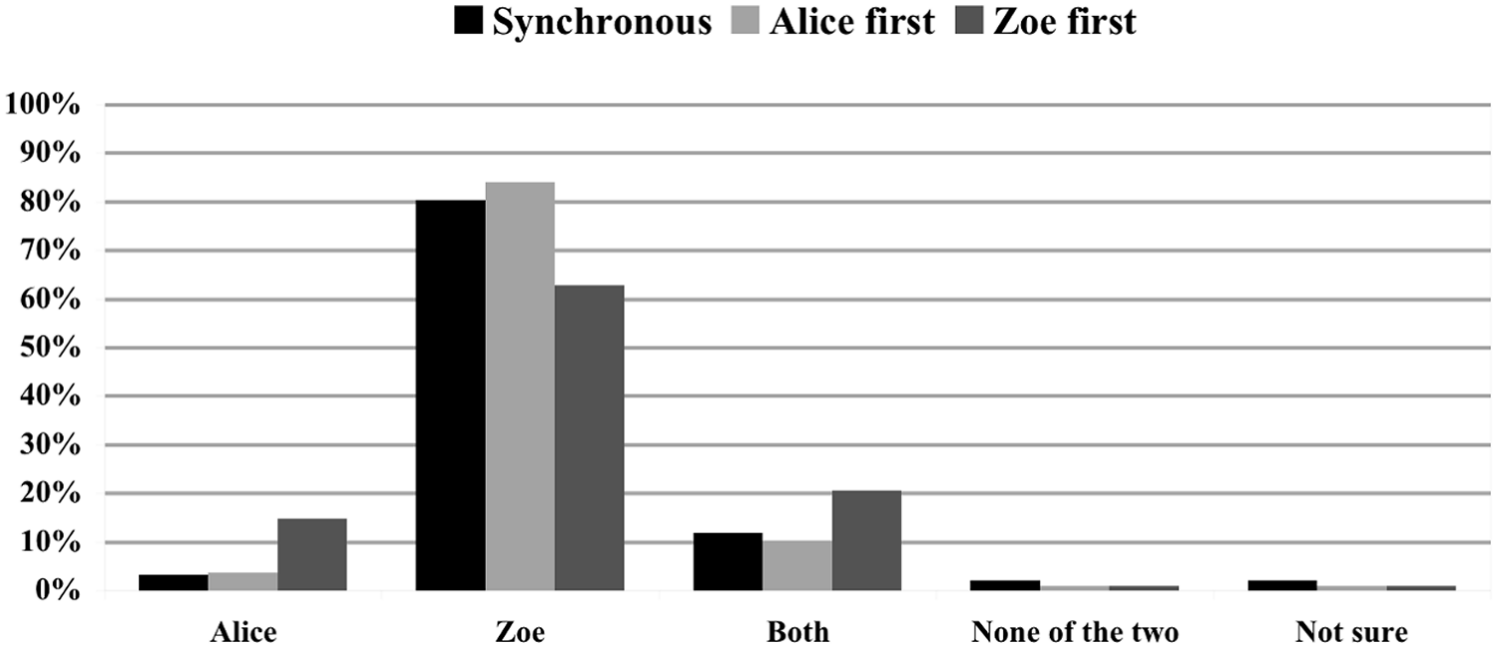
**Impact of temporal order on causal judgments in the rule violation condition.** Causal ratings in % for Scenario 10 (Alice and Zoe log in synchronously), Scenario 11 (Alice logs in first), and Scenario 12 (Zoe logs in first). The effect is held neutral.

The comparison between Scenario 10 (Alice and Zoe log in at the same time; neutral effect; Zoe violates a norm) and Scenario 11 (*N* = 107; Alice logs in first; neutral effect; Zoe violates a norm) did not yield any significant difference (χ^2^ = 1.493; *p* = 0.888), while the comparison between Scenario 10 and Scenario 12 (*N* = 102; Zoe logs in first; neutral effect; Zoe violates a norm) indicated that ‘Zoe’ responses dropped from 80.4 to 62.7%, ‘Alice’ responses increased from 3.3 to 14.7%, and ‘Both’ responses increased from 12.0 to 20.6% (see also **Figure 5**). This latter result (χ^2^ = 12.337; *p* = 0.007) was marginally significant after results were Bonferroni-corrected.

#### Effect × norm violation

**FIGURE 6:**
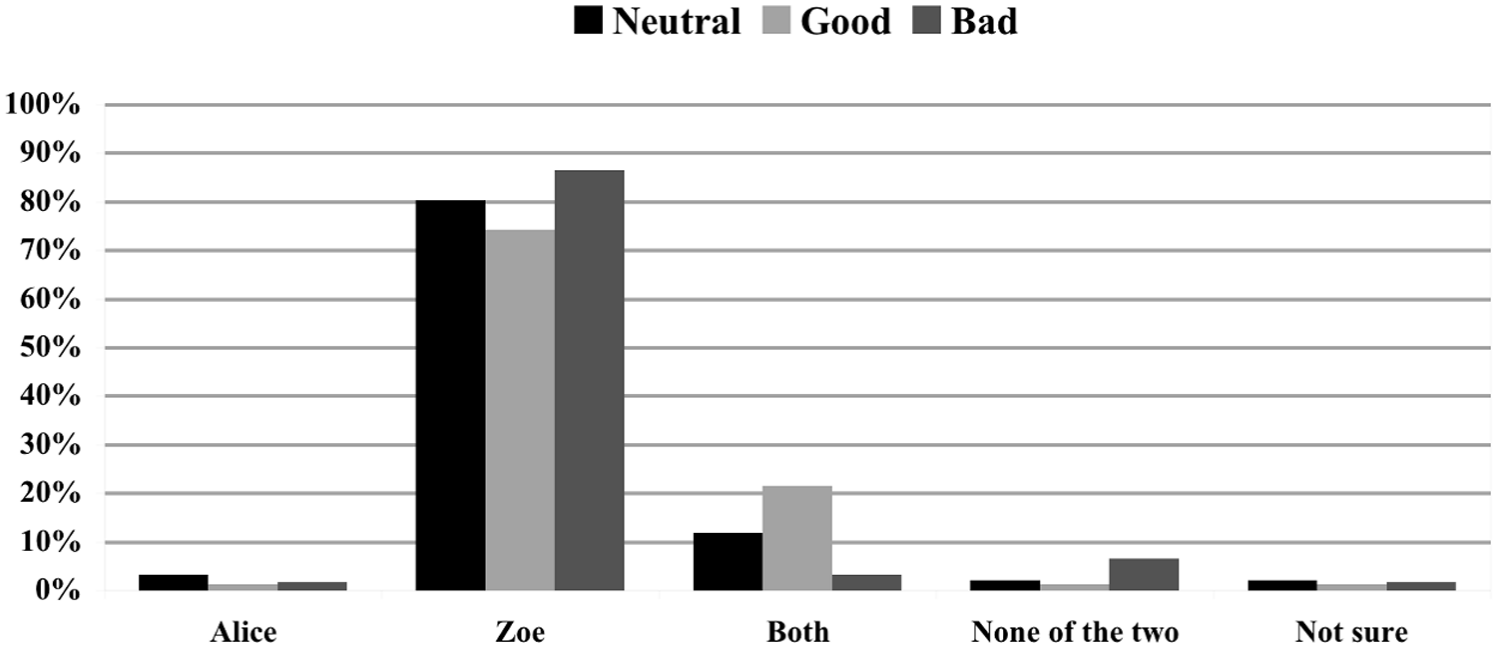
**Impact of the moral status of the effect on causal judgments in asynchronous, rule violation conditions.** Causal ratings in % for Scenario 10 (neutral effect), Scenario 13 (good effect) and Scenario 16 (bad effect).

To assess the impact of moral judgments about the effect on causal selection when a norm is violated, Scenarios 10 (Alice and Zoe log in at the same time; neutral effect; Zoe violates a norm), 13 (Alice and Zoe log in at the same time; good effect; Zoe violates a norm) and 16 (Alice and Zoe log in at the same time; bad effect; Zoe violates a norm) were compared (**Figure 6**). Compared to Scenario 10, the amount of people who considered Zoe to be the sole cause slightly decreased when a positive effect was presented and slightly rose for a negative effect. However, neither of these differences was significant (χ^2^ = 3.565, *p* = 0.500, and χ^2^ = 5.566, *p* = 0.202, respectively). The difference between Scenario 13 and Scenario 16 was marginally significant (χ^2^ = 12.359; *p* = 0.004) after Bonferroni correction.

### JUDGMENTS OF BLAME

For computing significant relationships in people’s attributions of blame in pairwise comparisons, Bonferroni-corrected Analysis of variance (ANOVA) was used. People rated the blame-/ praiseworthiness of Alice and Zoe on a 7-point Likert scale with ‘-3’ meaning ‘extremely blameworthy’ and ‘3’ meaning ‘extremely praiseworthy’.

**FIGURE 7:**
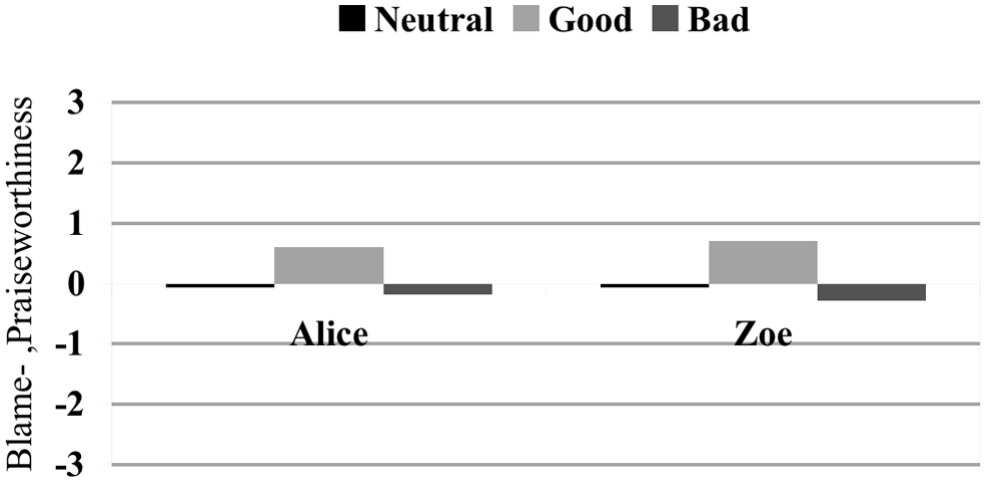
**Impact of the moral status of the effect on blame judgments.** Blame ratings on a 7-point Likert scale with ’-3’ meaning ‘extremely blameworthy’ and ‘3’ meaning ‘extremely praiseworthy’. Subjects rated the blame-/praiseworthiness of Zoe and Alice in scenarios without norm violation, with synchronous logging in, and with different effects.

**Figure 7** demonstrates that the values of blameworthiness of Alice and Zoe in Scenario 7 (Alice and Zoe log in at the same time; bad effect; no norm violation; 0.19, SD = 0.947; 0.30, SD = 0.954) did not significantly differ from Scenario 1 (Alice and Zoe log in at the same time; neutral effect; no norm violation; 0.06, SD = 0.639; 0.06, SD = 0.639), while subjects rated Alice and Zoe slightly praiseworthy in Scenario 4 (Alice and Zoe log in at the same time; good effect; no norm violation)^8^.

**FIGURE 8:**
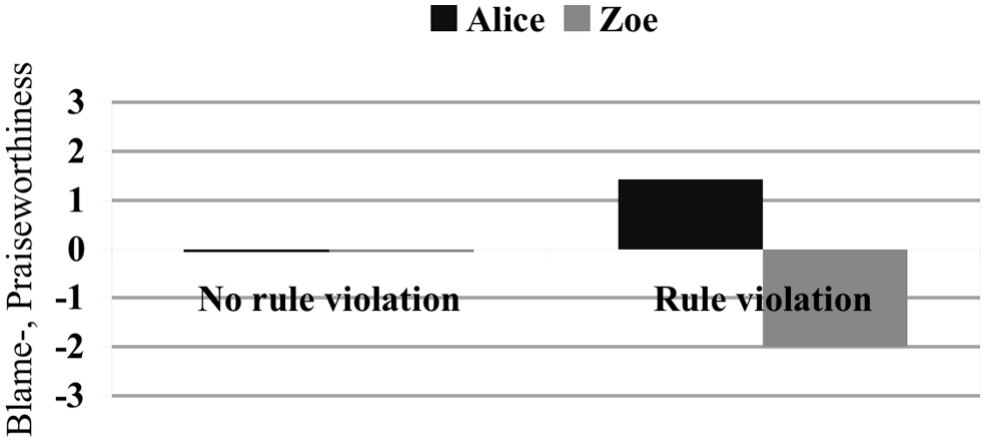
**Impact of rule violation on blame judgments.** Blame ratings on a 7-point Likert scale with ’-3’ meaning ‘extremely blameworthy’ and ‘3’ meaning ‘extremely praiseworthy’. Subjects rated the blame-/praiseworthiness of Zoe and Alice in scenarios with synchronous logging in, neutral effect, and different normative status.

In all scenarios in which no norm has been violated, people largely refrained from attributing praise or blame to either Alice or Zoe, even if a bad outcome had taken place. In contrast, as previous studies have already indicated (Hitchcock and Knobe, 2009; Alicke et al., 2011), norm violation seems to be an important factor for blame and praise attribution. As shown by **Figure 8**, not only do people strongly blame Zoe for having violated the rule (-2.00, SD = 1.075), they also consider Alice’s action to be in line with the policy of the company and hence high on praiseworthiness (+1,43, SD = 1.440)^9^.

## DISCUSSION

The first aim of our study was to assess whether and how the temporal location of human actions in a *temporal chain* influences judgments of actual causation. More precisely, we were interested in whether judgments of actual causation about two individuals performing the same kind of action A at the same time would differ from judgments of actual causation about a temporal chain in which two individuals perform A one after the other. Probabilistic models of the role of temporal location in causal selection predicted that subjects will tend to select both agents to be the actual cause in the synchronous condition, while they will tend to choose the last agent as actual cause in the asynchronous condition (Spellman, 1997). Our results support this prediction. To begin with, multinomial logistic regression revealed that temporal location is a significant predictor of judgments of actual causation. In particular, parameter estimates showed that, when two agents perform the same action one after the other, subjects are significantly more likely to select the last agent rather than both agents as the actual cause. In addition, comparisons between Scenario 1 (Alice and Zoe log in at the same time; neutral effect, no rule violation) and Scenarios 2 and 3 (Alice and Zoe log in at different times; neutral effect; no rule violation) indicated that while 81.5% of subjects selected both agents as actual causes in Scenario 1, only 39.5% of subjects selected both agents as actual causes in the asynchronous conditions and 43.9% of subjects selected the last agent as the actual cause (see **Figure 1**). Interestingly, our results differ from those obtained by McClure et al. (2007), who found that, when presented with two asynchronous actions in an *opportunity chain*, people tend to judge both actions to be actual causes. Further investigation is needed to establish why causal reasoning about temporal chains and opportunity chains differ with respect to the influence of temporal order.

The second aim of our study was to establish whether moral judgments about the effect influence causal selection even in the case in which agents could not have foreseen the effect and did not intend to bring it about. The NVA predicts that moral judgments about the effect would not impact on causal selection, given that this account “makes no mention of any sort of moral judgment regarding the effect” (Hitchcock and Knobe, 2009). The CCM instead predicts that moral judgments about the effect would influence actual causation as a function of the blame attributions that they generate. Importantly, both NVA and CCM fail to accommodate our results. *Pace* NVA, multinomial logistic regression revealed that the nature of the effect is a significant predictor of judgments of actual causation. In particular, parameter estimates indicated that judging the effect to be bad predicts the judgment that no one has caused the effect—in contrast, judging to effect to be good did not significantly alter judgments of actual causation with respect to judging the effect to be neutral. Moreover, as can be seen by the comparison between Scenario 1 (Alice and Zoe log in at the same time; neutral effect, no norm violation) and Scenario 7 (Alice and Zoe log in at the same time; bad effect, no norm violation), the influence of judgments about the effect is entirely independent from judgments of norm violation: in both scenarios no norm violation occurs, but in Scenario 7, in which a bad effect obtains, the amount of ‘Both’ responses dropped from 81.5 to 48.6%, whereas the percentage of ‘None of the two’ responses rose from 11.1 to 43.2% (see **Figure 2**).

However, CCM cannot explain these results either. According to it, judging an effect to be negative (rather than neutral) will trigger the desire to blame someone, and causal attributions will accordingly be automatically enhanced (with respect to a neutral condition) to “rationalize” this desire. There are two problems with this proposal. First, subjects considered Alice and Zoe to have causally contributed to the effect to a significantly lesser extent when the effect is negative (Scenario 7) than when it is neutral (Scenario 1); second, judgments of blameworthiness were the same in both scenarios (see **Figure 7**), hence CCM predicts that subjects’ judgments of actual causation should not be different in these two conditions.

Accordingly, our results can be summarized as follows: (i) moral judgments about the effect influence causal selection even in the case in which the effect was neither foreseen nor intended; (ii) the influence of moral judgments about the effect on causal selection cannot be reduced either to the influence of judgments of norm violation (*contra* NVA) or to an automatic process of blame validation (*contra* CCM); (iii) when an agent S performs an action A that is followed by an effect E, and S did not foresee or intend E, then subjects are significantly less disposed to judge that S caused E if they judge E to be negative (rather than neutral or positive). In order to explain (i)-(iii), one might propose that causal attributions are linked to attributions of moral responsibility (cf. Sytsma et al., 2012) and hypothesize the existence of an *anti-bad luck condition*, according to which, in order for one to be held responsible for a negative effect E, but not for a positive or neutral one, one should have intended or foreseen E. According to this account, when an unintended and unforeseen positive/neutral effect follows a certain action A performed by a certain subject S, people are willing to consider A as the cause of E since they are willing to hold S morally responsible for E; in contrast, when an unintended and unforeseen negative effect follows a certain action A performed by a certain subject S, people are reluctant to consider A as the cause of E, since the anti-bad luck condition prevents them to judge S to be morally responsible for E. In our study, however, we did not directly investigate subjects’ judgments of moral responsibility. Hence, further evidence is needed to assess an explanation of our results along these lines.

Our third aim was to establish whether judgments of norm violation influence causal selection even in the case in which the violated norm has nothing to do with the effect that obtains. One might expect that this would not be the case. After all, why should violating a norm N make a person more causally responsible for E if N and E are independent of each other? Things, however, are entirely different. First, multinomial logistic regression indicated that norm violation is by far the most significant predictor of judgments of actual causation: parameter estimates indicated that Zoe violating a norm hugely increases the likelihood of Zoe being judged to be the sole cause. Second, if a neutral effect followed the synchronous logging in of Alice and Zoe in the absence of any norm violation (Scenario 1), 81.5% of subjects answered that both Alice and Zoe caused the effect and only 11.1% answered that Zoe was the only cause. In striking contrast, when Zoe violated a norm in the neutral synchronous scenario (Scenario 10), 80.4% judged Zoe to be the sole cause and only 12.0% answered that both Alice and Zoe caused the effect (see **Figure 3**).

The role of judgments of norm violation in causal reasoning can be further appreciated by briefly considering the interplay between these judgments and judgments about temporal location, which has been the fourth aim of our study. Let’s consider the scenario in which Alice logs in later, Zoe violates a norm, and a neutral effect follows (Scenario 12). In this case, subjects are presented with elements that pull in opposite directions: on the one hand, the fact that Alice logged in later should incline subjects to answer that Alice caused the neutral effect; on the other hand, the fact that Zoe violated a norm should incline them to answer that Zoe caused the neutral effect. The cognitive conflict is solved in a way that shows the important role of norm violation in causal selection: even though Zoe acted before Alice, the fact that Zoe violated a norm is sufficient for 62.7% of subject to answer that she is the sole cause of the neutral effect (see **Figure 5**). A similar result emerges when analyzing the interplay between judgments of norm violation and judgments about the effect: when a rule was violated, the moral status of the effect had no significant impact on people’s judgments of causal selection (see **Figure 6**).

One might be tempted to consider our results as evidence in favor of Hitchcock and Knobe’s (2009) NVA, according to which judgments of norm violations play a pervasive and fundamental role in guiding causal attributions. Our findings, however, are not sufficient to conclude that NVA is the right explanation of the role of judgments of norm violation in causal selection—that is, they are not sufficient to conclude that judgments of norm violation influence causal selection *qua* judgments of norm violation. In fact, three alternative explanations can be provided. First, since we found that judgments of norm violation reliably trigger attributions of blame (**Figure 8**), our results can be explained by adopting Alicke’s (2000) CCM, according to which judgments of norm violation influence causal selection in virtue of triggering blame attributions. Second, if subjects tend to judge counternormative behaviors as statistically atypical, it may well be the case that the judgment that Zoe’s behavior was a norm violation suggested to subjects that Zoe’s behavior was less statistically typical than Alice’s, and it was this latter judgment that influenced the subjects’ causal selection processes (Driver, 2008; but see Knobe and Fraser, 2008 for a rejoinder). Third, if subjects consider counternormative behaviors to happen less often than normative ones, subjects might have judged that Zoe’s behavior covaried with the effect (i.e., they might have judged that it was the first time that Zoe logged in to the computer and also the first time that the effect occurred), and covariation between an event and an effect is known to have a huge impact on causal judgments (Cheng and Novick, 1992). Further evidence is needed to adjudicate among these hypotheses.

In conclusion, our study established that temporal location, the moral status of the effect, and norm violation are all significant predictors of judgments of actual causation. In line with probabilistic models of temporal location, we showed that, when presented with a temporal chain in which two human agents perform the same action one after the other, subjects tend to judge the later agent to be the actual cause. This effect, however, is significantly weakened if the second agent did not violate a norm and the first did, an effect that is predicted by NVA, but can also be explained by CCM. However, neither of these theories can account for the role of moral judgments about the effect in causal reasoning.

## Conflict of Interest Statement

The authors declare that the research was conducted in the absence of any commercial or financial relationships that could be construed as a potential conflict of interest.

## Footnotes

1 More precisely, we conceive of a temporal chain as follows: a temporal chain from x_1_ to effect y consists of a set of causes x_1_, x_2_,… x_n_ such that (i) x_1_, x_2_,… x_n_ together cause y; (ii) x_1_ is prior to x_2_, x_2_ is prior to… x_n;_ (iii) x_1_, x_2_,… x_n_ are not otherwise causally related; (iv) it is not the case that x_n_ has to be expected on the basis of x_n-1_.

2 Again, a more precise characterization of the notion of an unfolding causal chain would run like this: an unfolding causal chain from x_1_ to effect y consists of a set of causes x_1_, x_2_,… x_n_ such that (i) x_1_, x_2_,… x_n_ together cause y; (ii) x_1_ is prior to x_2_, x_2_ is prior to… x_n_; (iii) each successive cause is caused by all its causal predecessors (e.g., x_2_ is caused by x_1_ and x_3_ is caused by x_1_ and x_2_); (iv) x_n_ has to be expected on the basis of its causal predecessors.

3 It is not an easy task to distinguish opportunity causal chains from unfolding causal chains. For our purposes, the following partial account of the notion of an unfolding causal chain will do: an opportunity chain from x_1_ to effect y consists of a set of causes x_1_, x_2_,… x_n_, z such that (i) x_1_, x_2_,… x_n,_ and z together cause y; (ii) x_1_ is prior to x_2_, x_2_ is prior to… x_n;_ (iii) y is not be expected on the basis of x_1_-x_n,_ and y is to be expected on the basis of z and x_1_-x_n_.

4 One might wonder whether reducing the impact of morality on causal judgments to the impact of normality on causal judgments does not in fact reduce the impact of morality on causal judgments to purely statistical considerations, thus undermining Knobe’s (2010) idea that causal reasoning about human actions is not sensitive to statistical information only. The answer is negative, given that, according to Hitchcock and Knobe (2009), normality in the moral sense is distinct from normality in the statistical sense.

5 We developed these scenarios on the basis of a vignette used by Knobe (2006).

6 We verified the moral status of the different outcomes of the scenarios by asking 79 subjects about the status of the outcome when no rule had been violated, and 84 subjects about the status of the outcome when a rule had been violated. Each subject only received a single question in order to avoid comparative evaluations, e.g., ‘How would you evaluate the fact that some spam emails containing dangerous viruses are deleted from the central computer on a scale from ‘-3’ meaning ‘Very bad’ to ‘3’ meaning ‘Very good’?’ Results are as follows. No rule violation: neutral outcome = -0.54 (SD = 0.89); good outcome = 2.27 (SD = 1.20); bad outcome = -2.42 (SD = 0.92). Rule Violation: neutral outcome = -0.56 (SD = 0.99); good outcome = 2.00 (SD = 1.11); bad outcome = -2.38 (SD = 0.73).

7 Note that Lagnado and Channon (2008) and Alicke et al. (2011) are insensitive to the distinction between causal reports and causal explanations and treat, at least in wording, causal explanation and causal reports on a par. McClure et al. (2007) and Hilton et al. (2009) explicitly phrase the question in terms of selection of causal explanations; and McClure et al. suggest that what is tested for is the quality of an explanation which is, as they put it, “strongly related to […] communicative relevance,” rather than something like “causal importance” (McClure et al., 2007, 892).

8 ANOVA: Alice: *F*(2,138) = 10.393, *p* < 0.001. Zoe: *F*(2,138) = 15.564, *p* < 0.001. A *Post hoc* test revealed no significant difference between neutral and bad condition: Alice: *p* = 1.000, Zoe: *p* = 0.611.

9 ANOVA: Alice: *F*(1,91) = 42.378, *p* < 0.001; Zoe: *F*(1,91) = 111.999, *p* < 0.001.
